# Ovarian primary primitive neuroectodermal tumor: a review of cases at PUMCH and in the published literature

**DOI:** 10.1186/s13023-019-1106-5

**Published:** 2019-06-19

**Authors:** Xiaopei Chao, Yalan Bi, Lei Li

**Affiliations:** 1 Department of Obstetrics and Gynecology, Peking Union Medical College Hospital, Shuaifuyuan No. 1, Dongcheng District, Beijing, 100730 China; 2 Department of Pathology, Peking Union Medical College Hospital, Beijing, 100730 China

**Keywords:** Ovary tumor, Primary neuroectodermal tumor, Chemotherapy, Radiotherapy, Target therapy, Prognosis

## Abstract

**Background:**

The pathological characteristics, treatment strategies and prognosis of ovarian primary primitive neuroectodermal tumor (PNET) were unclear due to the rarity of PNET. All cases treated at Peking Union Medical College Hospital (PUMCH) between 1975 and 2016 and published in the English literature between 1980 to 2017 were reviewed.

**Results:**

Finally four cases from PUMCH and 15 cases in the literature were included. The median age was 25 years (range 13–79), and the median diameter of the tumors was 13.4 cm (range 5.0–30.0). The most common initial symptoms were abdominal pain, bloating and a pelvic mass. Diagnosis primarily depended on immunohistochemical and fluorescence in situ hybridization data. Treatment consisted of surgery, various chemotherapy regimens and/or radiotherapy. The 5-year overall survival (OS) and progression-free survival (PFS) rates were 15 and 52%, respectively. For patients with OS and PFS > 12 months, the median ages were 21 years (range 13–35) and 17 years (range 13–35), respectively, while for patients with OS < 12 months and PFS < 12 months, the median ages were 48 years (range 14–79) and 25 years (range 18–79), respectively.

**Conclusions:**

No standard therapy for ovarian primary PNET exists, and an individualized strategy is recommended. Young patients seem to have better prognoses.

## Introduction

**Fig. 1:**
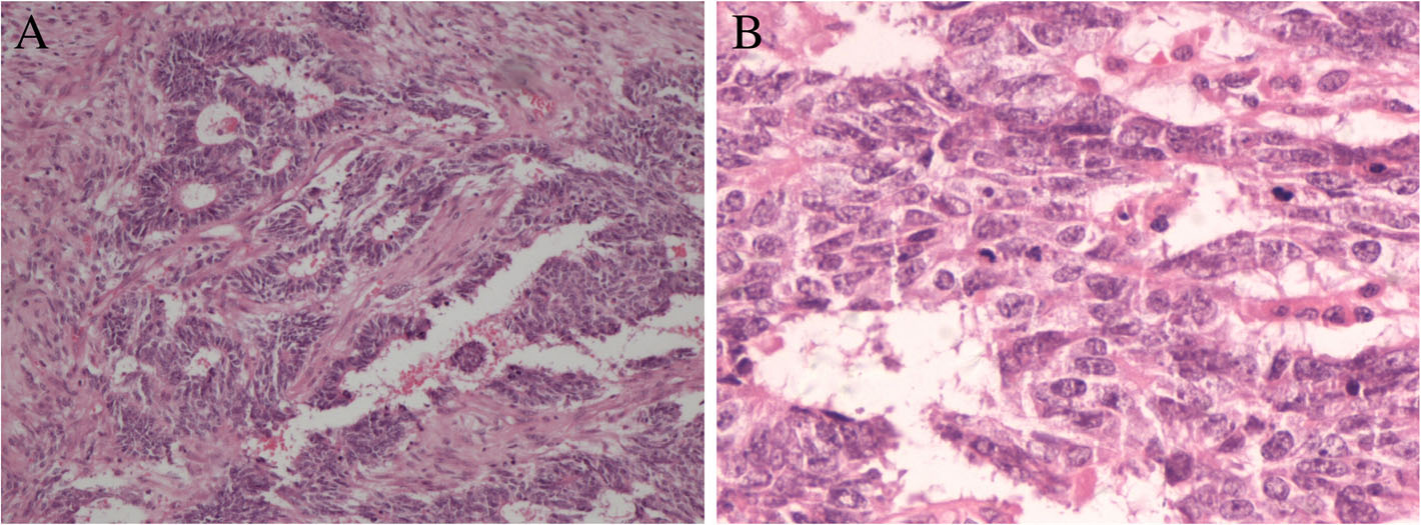
Pathological sections of hematoxylin and eosin revealed that ovarian primary PNET comprises a homogeneous population of small- to medium-sized round primitive cells that grow in sheets, nests and cords. They display high nuclear-to-cytoplasmic ratios and brisk mitotic activity. Figures are from case 3 (description refers to the main text). **a** 100 × . (**b**) 400×

Primitive neuroectodermal tumor (PNET) was first proposed by Hart and Earle [1] in 1973. PNET is classified as central PNET and peripheral PNET (pPNET), and the most common sites of the latter are the paravertebral region and the chest wall. The most common site of pPNET in the female genital tract is the ovary [2], followed by the uterine corpus [2–7]; in contrast, pPNET in the cervix and vulva is extremely rare [2, 7]. Classification of pPNET depends primarily on the differentiation of its neural components. According to World Health Organization (WHO) classification, ovarian primary PNET can be divided into either tumors that resemble their central nervous system counterparts or pPNET/Ewing’s sarcomas. Most tumors comprise a homogenous population of small- to medium-sized round primitive cells that grow in sheets, nests and cords. They display high nuclear-to-cytoplasmic ratios and vigorous mitotic activity (Fig. 1) [8]. However, because of the rarity of PNET, no standard treatment plans exist to date, and the clinical and tumor characteristics and prognosis have not been clearly described. In this study, we report four cases of ovarian primary PNET treated at Peking Union Medical College Hospital (PUMCH), Beijing, China, between 1975 and 2016 and 15 cases reported in the English literature between 1980 and 2017. Clinicopathological and survival data were collected and analyzed.

## Materials and methods

### Ethics approval and consent

All procedures performed in the study involving human participants were in accordance with the ethical standards of the institutional and/or national research committee and with the 1964 Declaration of Helsinki and its later amendments or comparable ethical standards. All patients or their authorized deputies had presented consents. The Institutional Review Board of PUMCH had approved this study (ID HS-1390).

### Cases in Peking union medical college

Through an intensive search of the database of electronic medical records from PUMCH from 1975 to 2016, four cases of ovarian primary PNET were retrieved. The pathological results were verified by Dr. Bi. None of these cases has been published before.

#### Case 1

A 33-year-old woman presented with complaints of progressive backache and lower limb aching 7 months after laparoscopic right ovarian cystectomy, the pathology of which proved to be mature teratoma. Sonographic examination revealed a mass in the right iliac. Single-photon emission computed tomography showed a malignant metastatic lesion in the hip bone, and pathological review of the first surgical section suggested the possibility of an immature teratoma. Despite 11 courses of cisplatin-based combination chemotherapy and two courses of radiotherapy, the disease progressed during treatment, with multiple metastases in the right iliac fossa, iliac, sacrum and liver on imaging. Biopsy of the liver lesion proved it to be a metastasis. Subsequent palliative radiotherapy did not relieve her pain. Palliative surgery was performed with partial hepatectomy of the left lobe of the liver and resections of the omentum and metastatic lesions. The final pathological exam showed metastatic PNET from the ovaries. The patient declined further treatment and died 2 years after the bone metastasis.

#### Case 2

A 59-year-old woman had undergone exploratory laparotomy and pelvic mass resection with elevated CA-125 (105 U/ml). Pathology suggested a lipoid cell tumor. The pelvic mass recurred 3 months after the primary surgery, and a second laparotomy revealed multiple solid tumors in the pelvic and abdominal cavities. Tumor cytoreductive surgery was performed with total hysterectomy, bilateral salpingo-oophorectomy, appendectomy, infracolic omental excision, and metastatic lesion resection, with a residual tumor less than 1 cm. The final pathological diagnosis was primary ovarian PNET. Immunohistochemical (IHC) staining showed a positive reaction for cluster of differentiation (CD)-99, neuron-specific enolase (NSE), vimentin and smooth muscle actin (SMA) but was negative for synaptophysin, alpha fetoprotein (AFP), epithelial membrane antigen (EMA), calretinin, a-inhibin, Melan A, and desmin. The patient refused further adjuvant chemotherapy and was lost to follow-up.

#### Case 3

**Fig. 2:**
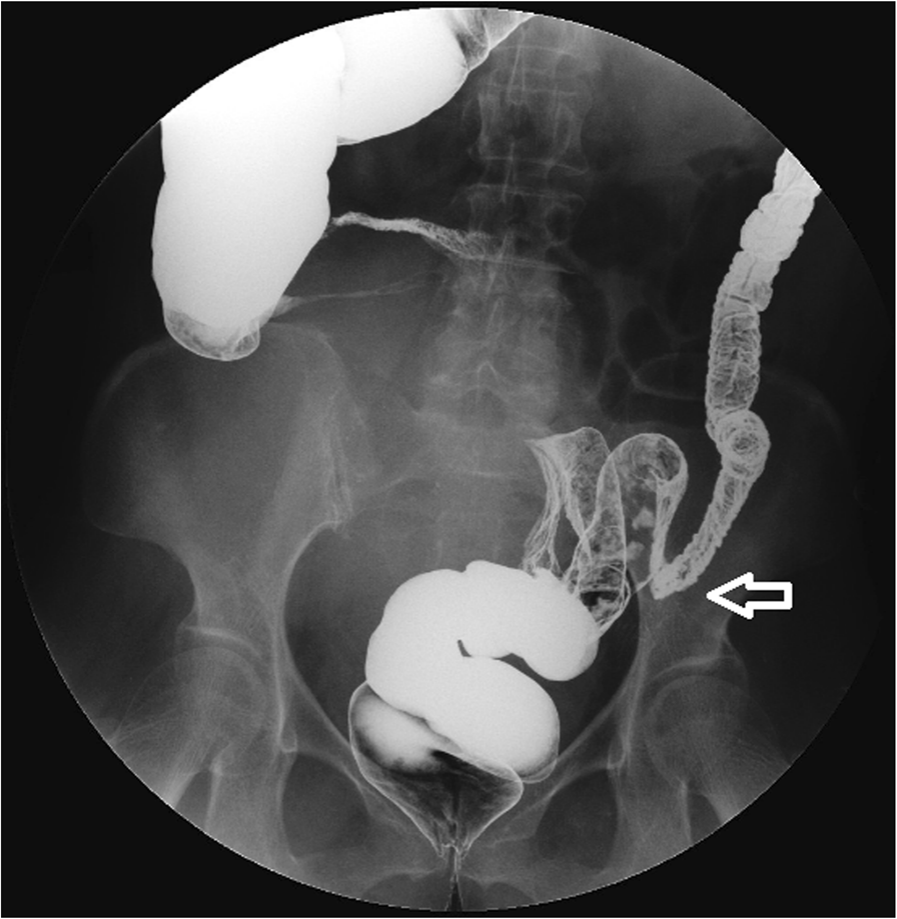
Colonoscopy and colonography in case 3 (description refers to the main text) indicates sigmoid colon adhesions and stenosis (arrow)

**Fig. 3:**
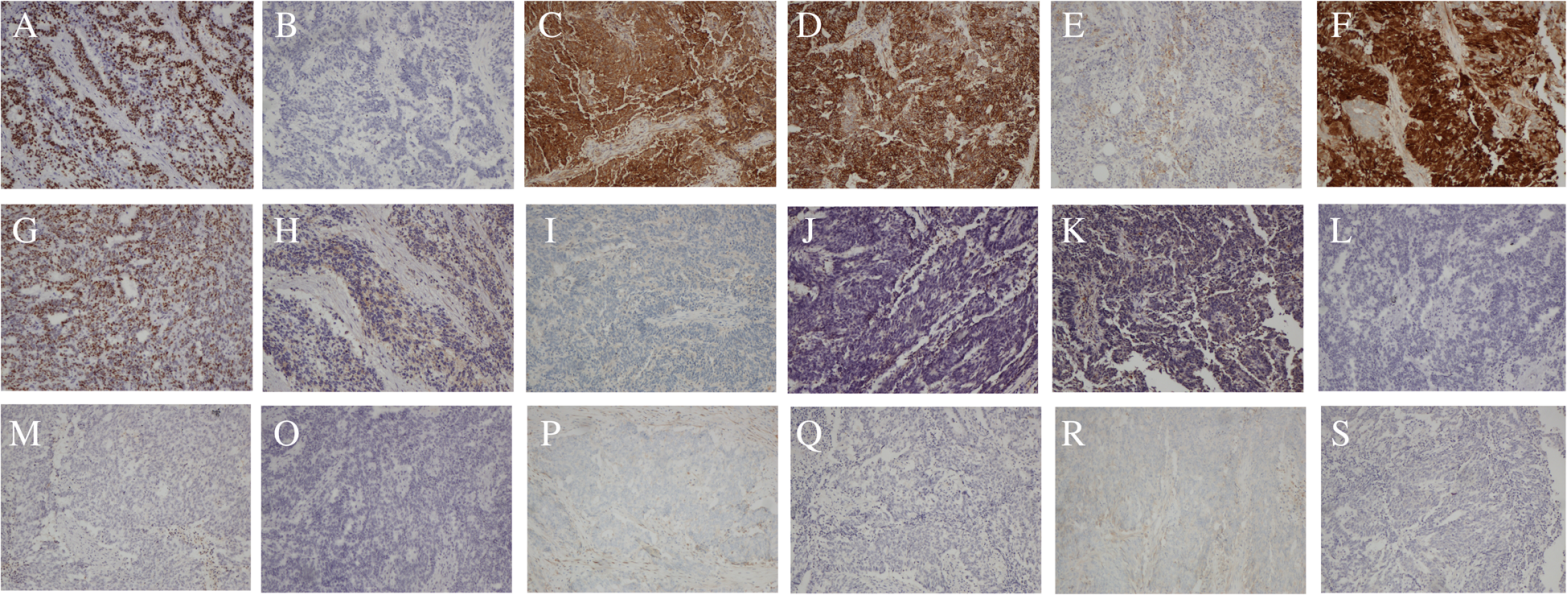
Immunohistochemical staining results in case 3 (description refers to the main text). Immunohistochemical staining reveals positive staining for (**a**) Ki-67 (labeling index, 50%), **b** neurofilament (focal+), **c** NSE, (D) nestin, **e** synaptophysin (focal+), **f** P16 and (**g**) P53 but negative staining for (**h**) CD-99, **i** glial fibrillary acidic protein (GFAP), **j** octamer-binding transcription factor (OCT) 3/4, **k** S-100, **l** estrogen receptor (ER), **m** paired box gene 8 (PAX-8), **o** progesterone receptor (PR), **p** wilms tumor (WT)-1, **q** creatine kinase (CK)20, **r** CK7 and (**s**) calretinin

A 67-year-old woman presented with abdominal distention and changes in bowel habits that had occurred over the previous 4 months. She had undergone transabdominal hysterectomy and left salpingo-oophorectomy for some benign diseases 2 years prior. Colonoscopy and colonography indicated sigmoid colon adhesions and stenosis (Fig. 2). Ultrasonography revealed a smooth-surfaced pelvic mass of approximately 22 cm in diameter. Tumor marker analysis revealed elevated CA-125 (104.2 U/ml). Tumor cytoreductive surgery was performed with right salpingo-oophorectomy, appendectomy, infracolic omental excision, and partial excision of the sigmoid colon and small intestine. IHC analysis revealed positive staining for Ki-67 (labeling index, 50%), neurofilament (focal+), NSE, nestin, synaptophysin (focal+), P16 and P53 but was negative for CD-99, glial fibrillary acidic protein (GFAP), octamer-binding transcription factor (OCT) 3/4, S-100, estrogen receptor (ER), paired box gene 8 (PAX-8), progesterone receptor (PR), Wilms tumor (WT)-1, creatine kinase (CK)20, CK7 and calretinin (Fig. 3). The final pathological diagnosis was primary ovarian PNET extending to the serosa of the rectum, sigmoid colon, appendix and small intestine as well as the lymph nodes around the colon. The disease was stage III according FIGO criteria [9]. The patient refused further treatment and died 6 months after her initial symptoms were observed.

#### Case 4

**Fig. 4:**
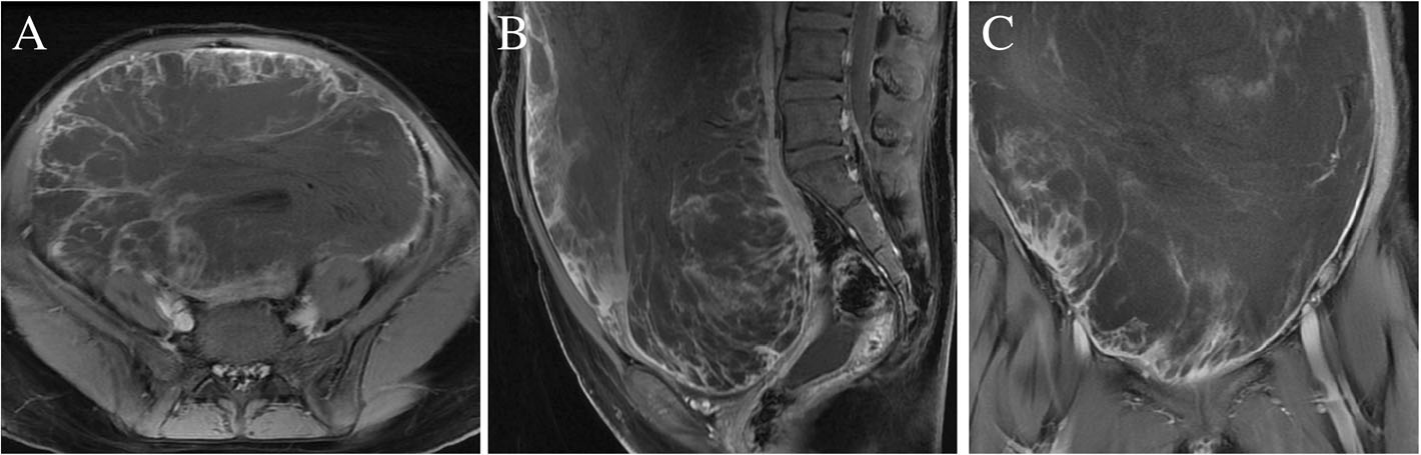
T2 Magnetic resonance imaging with enhancement of the pelvic mass in case 4 (description refers to the main text). **a** Axial view. **b** Sagittal view. **c** Coronal view

A 14-year-old girl presented with abdominal distention for 2 weeks, which worsened over 1 week. Sonographic examination revealed a 30 cm × 20 cm pelvic mass with the possibility of ovarian origin. Abdominal and pelvic magnetic resonance imaging (MRI) revealed a large mass in the abdominal and pelvic cavity, which showed a low and intermediate T1 signal intensity and a high, heterogeneous T2 signal intensity, with good enhancement (Fig. 4). Analysis of tumor markers revealed elevated CA-125 (473.0 U/ml), lactate dehydrogenase (LDH, 682 U/l) and NSE (18.2 ng/ml); levels of AFP, CA-199, CEA and β-human chorionic gonadotropin were normal. Laparotomy revealed a large solid mass, measuring approximately 30 cm in diameter and originating from the right ovary, and an omental mass measuring approximately 5 cm in diameter. Frozen tissue sections suggested a poorly differentiated carcinoma. Resection of the right annex, biopsy of the left ovary, omentectomy, and appendectomy were carried out, with a residual tumor less than 1 cm. IHC staining showed a positive reaction for CD99 but negativity for synaptophysin, calretinin, CD30 (Ki-1), CD10, cytokeratin (AE1/AE3), S-100 and vimentin. The final pathological diagnosis was primary ovarian PNET with diffuse metastasis to the omental and Douglas cavity. The disease was stage IIIC according to the FIGO criteria. Adjuvant chemotherapy was administered with paclitaxel (175 mg/m^2^) plus carboplatin (AUC 6). Contrast-enhanced computed tomography of the abdomen and pelvis revealed tumor recurrence during the course of chemotherapy. The patient died of disease progression; her overall survival (OS) time was 5 months.

### Review of the literature

A search of the English-language literature from January 1980 to December 2017 was performed in PubMed, EMBASE and Google Scholar using the following key words: “primitive neuroectodermal tumor”, “Ewing’s sarcoma” and “neuroectodermal-type tumor”. Ultimately, 12 reports comprising 15 cases with detailed clinical data were included for analysis [10–21].

### Statistics

The cases from PUMCH and previous reports were pooled for analysis of clinicopathological characteristics. Some survival data were collected through correspondence with authors (Dr. Kyong-Jin Kim, Dr. Bharat Rekhi and Dr. Kawauchi, Shigeto) by private communication. SPSS 23.0 (SPSS Inc., Chicago, IL, USA) was applied for statistical analysis. Life tables were used to determine 1-, 3- and 5-year OS rates.

## Results

The clinicopathological data and results of IHC and molecular genetics analysis are shown in Tables 1 and 2.

**Table 1:** Clinicopathological data of 19 cases with ovarian PNETs

Author	Age (Y)	Clinical manifestation	Tumor size	Stage	Treatment	Outcome	OS
Aguirre, 1982 (Aguirre and Scully 1982) [10]	14	Increased AC, dyspnea, AUB	R/20 × 15 cm, 1278 g	IIIB^a^	RSO + OM + appendectomy; RAD	Progression	DOD, 2 M
Aguirre, 1982 (Aguirre and Scully 1982) [10]	13	Abdominal pain and distention after RSO+ appendectomy	8 × 5 cm	III^a^	Suboptimal CRS (LSO+ pelvic mass resection); melphalan×1; (CTX + KSM) × 2	Progression	DOD, 20 M
Aguirre, 1982 (Aguirre and Scully 1982) [10]	16	Abdominal pain; family history of tumor	L/15 cm, 464 g	IA	LSO+ appendectomy; TAH + RSO 6 days later; no adjuvant therapy	Relapse, 36 M	DOD, 47 M
Aguirre, 1982 (Aguirre and Scully 1982) [10]	17	Pelvic mass	L/18 cm	IA	LSO + ROV biopsy; no adjuvant therapy	Complete remission	NED, 84 M
Kanbour-Shak, 1993 (Kanbour-Shakir et al. 1993) [11]	35	Abdominal distention and increased AC for 2 M	R/18 × 10 × 10 cm	IIIA^a^	Complete surgical staging (TAH + RSO + LS (history of LOV resection) + OM + PLND+PALND); BEP × 6	Complete remission	NED, 42 M
Lawlor, 1997 (Lawlor et al. 1997) [12]	13	Intermittent fever, abdominal pain and diarrhea	Ovarian tumor 1448 g, omentum tumor 1855 g	IIIC^a^	Suboptimal CRS (partial resection) (RSO + OM); (DDP + VP16 + doxorubicin+CTX) Q4W × 4, PBPC	Complete remission	NED, 18 M
Kawauchi, 1998 (Kawauchi et al. 1998) [13]	29	Pelvic mass	L/22 × 17 × 15 cm; R/7 × 7 × 5 cm	IIIC^a^	Optimal CRS (without any residual disease) (TAH + BSO + OM + PALN biopsy); CHEM×12	Relapse	DOD, 11 M
PUMCH case 1, 2003^b^	33	Backache and lower limb aching	12.0 × 8.4 cm	IV	CHEM×11; RAD×2; PEB × 2; PVB × 1; RAD× 1; palliative surgery	Progression	DOD, 24 M
Demirtas, 2004 (Demirtas et al. 2004) [14]	25	Abdominal pain and distention for 3 M	L/8 cm	IC1	Fertility-sparing complete surgical staging (LSO + OM + partial ROV + PLND+PALND); BEP × 4; VIP × 6 when recurrent	Relapse, 3 M	NED, 28 M, CS twice
Chow, 2004 (Chow et al. 2004) [15]	13	Abdominal pain and distention for 1 M	N/A	IIIC^a^	Optimal CRS (without any residual disease) (BSO + OM + partial bladder); PEB × 2; RCRS and RAD	Progression	DOD, 17 M
Kim, 2004 (Kim et al. 2004) [16]	18	Pelvic mass	16 × 13 cm	IIIA^a^	Optimal CRS (without any residual disease) (RSO + Partial LOV + OM + PLN/PALN biopsy); TC × 6; RAD when recurrent; OP when intestinal obstruction; VACA	Relapse, 4 M	Died of septic shock, 10 M
PUMCH case 2, 2005^b^	59	Pelvic mass	N/A	III^a^	Optimal CRS (with residual tumor less than 1 cm)	N/A	N/A
Fischer, 2006 (Fischer et al. 2006) [17]	79	Abdominal distention for 4 M	6 × 4 cm	III	Optimal CRS (TAH + BSO + OM + rectum and sigmoid colon resection+ partial bladder+ peritoneum+ PALND); CHEM	Complete remission	NED, 6 M
Ateser, 2007 (Ateser et al. 2007) [18]	28	Stomach ache, nausea and vomiting at 12 weeks of gestation	25 × 30 cm	N/A	RSO; doxorubicin+CTX + VCR; (at 37 weeks of gestation) CS + Optimal CRS (without any residual disease) (TAH + LSO + OM+ metastatic lesion); altered with VACA and PEI, RAD	Progression	DOD, 13 M
Anfinan, 2009 (Anfinan et al. 2008) [19]	31	Abdominal pain and increased AC	15 × 12 × 10 cm	IIIC^a^	Optimal CRS (without any residual disease) (TAH + BSO + OM); Altered with VID and VIA; docetaxel+DDP	Progression	DOD, 15 M
Ostwal, 2012 (Ostwal et al. 2012) [20]	28	Pelvic mass for 2 M	12 × 11 × 10 cm	N/A	LOV resection; CHEM (Altered with VIE, VAC and VCD for 2 courses); CRS when recurrent	Relapse, 18 M	DOD, 18 M
Chu, 2014 (Chu et al. 2014) [21]	16	Pelvic mass	16.5 × 9.2 cm	IC	Fertility-sparing complete surgical staging (LSO + LPLND+OM); TC × 6	Complete remission	NED,13 M
PUMCH case 3, 2014^b^	67	Abdominal distention for 4 M	N/A	III^a^	Optimal CRS (without any residual disease) (history of TAH + LSO) (RSO + OM + appendectomy+partial small intestine and sigmoid colon resection); without adjuvant therapy	Progression	DOD, 6 M
PUMCH case 4, 2016^b^	14	Abdominal distention for one week	30 cm	IIIC	Optimal CRS (without any residual disease) (RSO + LOV biopsy+ OM + appendectomy+metastasis); TC × 1	Progression	DOD, 5 M

Abbreviations: *AC* Abdominal circumference, *AUB* Atypical uterine bleeding, *BSO* Bilateral salpingo-oophorectomy, *CHEM* Chemotherapy, *CRS* Cytoreductive surgery, *CS* Cesarean section, *CTX* cyclophosphamide, *DDP* cisplatin, *DOD* died of disease, *EP + THP* Etoposide, cisplatin, pirarubicin, *EP + TPT* etoposide, cisplatin, topotecan, *EWS* Ewing’s sarcoma, *IFO* Ifosfamide, *KSM* actinomycin, *L* Left side, *LOV* Left ovary, *LPLND* Left pelvic lymph node dissection, *LS* Left salpingectomy, *LSO* Left salpingo-oophorectomy, *M* Months, *N/A* Not available, *NED* No evidence of disease, *OM* Omentectomy, *OP* Operation, *OS* Overall survival, *PAC* Cisplatin, actinomycin, cyclophosphamide, *PALND* Paraaortic lymph node dissection, *PBPC* Peripheral Blood Progenitor Cell, *PEB* Cisplatin, etoposide, bleomycin, *PEI* Cisplatin, etoposide, ifosfamide, *PLND* Pelvic lymph node dissection, *PUMCH* Peking Union Medical College Hospital, *PVB* Cisplatin, vincristine, bleomycin, *R* Right side, *RAD* Radiotherapy, *RCRS* Recytoreductive surgery, *ROV* Right ovary, *RSO* Right salpingo-oophorectomy, *TAH* total abdominal hysterectomy, *TC* Paclitaxel, carboplatin, *VAC* Vincristine, actinomycin, cyclophosphamide, *VACA* Vincristine, actinomycin, cyclophosphamide, doxorubicin, *VCD* Vincristine, cyclophosphamide, doxorubicin, *VCR* Vincristine, *VIA* Vincristine, ifosfamide and actinomycin, *VID* Vincristine, ifosfamide and doxorubicin, *VIE* Vincristine, ifosfamide, etoposide, *VIP* Vincristine, ifosfamide, cisplatin, *VP16* etoposide, *Y* years

^a^at least this FIGO stage

^b^These cases are reported in this study

**Table 2:** The immunohistochemical and molecular genetic analysis of 14 cases

Case	IHC	Karyotype, t (11,22)	Molecular genetic analysis
Kanbour-Shakir, 1993 (Kanbour-Shakir et al. 1993) [11]	NSE(+), EMA(+)	N/A	N/A
Lawlor, 1997 (Lawlor et al. 1997) [12]	CD99(−), NSE(+)	–	EWS/FLI1, EWS/ERG gene fusions (−); MYCN gene amplification (−)
Kawauchi,1998 (Kawauchi et al. 1998) [13]	CD99(+)	+	EWS/FLI1(+);
PUMCH Case 1, 2003^a^	CD99(−)	N/A	N/A
Chow, 2004 (Chow et al. 2004) [15]	CD99(+), NSE(+)	N/A	N-myc, EGFR, FASL, GITRL overexpression, ARHI, FAT low expression
Kim, 2004 (Kim et al. 2004) [16]	CD99(+), FLI-1(+)	N/A	N/A
PUMCH Case 2, 2005^a^	CD99(+), NSE(+)	N/A	N/A
Fischer, 2006 (Fischer et al. 2006) [17]	CD99(+), NSE(+), FlI-1(+)	N/A	N/A
Ateser, 2007 (Ateser et al. 2007) [18]	CD99(+)	N/A	N/A
Anfinan, 2009 (Anfinan et al. 2008) [19]	CD99(+), NSE(+)	N/A	N/A
Ostwal,2012 (Ostwal et al. 2012) [20]	CD99(+), FLI-1(+)	+	EWS/FLI1(+); EWS/WT1(−)
Chu, 2014 (Chu et al. 2014) [21]	CD99(−)	N/A	N/A
PUMCH Case 3, 2014^a^	CD99(−), NSE(+)	N/A	N/A
PUMCH Case 4, 2016^a^	CD99(+)	N/A	N/A

Abbreviations: *CD99* Cluster of differentiation 99, *EGFR* Epidermal growth factor receptor, *EMA* Epithelial membrane antigen, *FLI-1* Friend leukemia integration 1 transcription factor, *GITRL* Glucocorticoid-induced tumor necrosis factor receptor ligand, *IHC* Immunohistochemistry, *N/A* Not available, *N-myc* N-myc proto-oncogene protein, *NSE* Neuron-specific enolase

^a^These cases are reported in this study

For all 19 cases, the median age of the patients was 25 years (range 13–79), and most of them (16/19, 84.2%) were premenopausal girls and women. The clinical manifestations were abdominal pain and/or distention (11 cases, 57.9%), a pelvic/abdominal mass (6 cases, 31.6%), irregular menstruation (1 case, 5.3%), and progressive backache and lower limb aching (1 case, 5.3%). The lesions were all unilateral with a median diameter of 13.4 cm (range 5.0–30.0). Of the 16 cases with exact FIGO stages, 3 (18.8%), 12 (75.0%) and 1 (6.3%) were FIGO stage I, III and IV, respectively. Except for one patient with bone metastasis, 18 underwent primary surgeries: 13 patients received tumor cytoreductive surgery or comprehensive staging, and 5 patients received non-comprehensive staging surgery. Overall, four patients accepted fertility-sparing surgeries.

Eleven of the 19 cases (57.9%) were treated with different types of chemotherapy regimens: 4 patients received chemotherapy prescribed for germ cell tumors, such as PEB (cisplatin, etoposide, bleomycin), VIP (vincristine, ifosfamide, cisplatin), VID (vincristine, ifosfamide and doxorubicin) or VIA (vincristine, ifosfamide and actinomycin); 3 accepted the TC regimen (paclitaxel, carboplatin); one underwent the EFT-2001 strategy prescribed for Ewing sarcoma family tumor (ESFT); one was prescribed melphalan, actinomycin and cyclophosphamide; and 2 were treated with unknown chemotherapy regimens. Three patients received a combination of chemotherapy and radiotherapy after surgery, and only one patient received simple adjuvant radiotherapy after surgery.

IHC and/or molecular genetic analyses were performed for 13 of the 19 cases (68.4%). The most common positive IHC markers were CD99 (9/12, 75%), NSE (7/7, 100%) and FLI-1 (friend leukemia integration 1 transcription factor) (3/3, 100%). Molecular genetic analyses were conducted for 3 cases: two showed the chromosomal translocation t (11;22); for the other case, EWS/FLI-1 chimeric mRNA was detected by reverse transcription-polymerase chain reaction (RT-PCR).

After primary treatment, definite survival data were available for 18 of the patients. Eight of 18 (44.4%) experienced progressive disease and died, whereas 10 patients achieved complete remission (CR). However, 5 patients experienced tumor relapse (4 cases died, and one achieved CR), and 5 patients remained in CR. The median OS rate was 18.0 months (for 18 patients, range 2.0–84.0), and the 1-, 3- and 5-year OS rates were 65, 31 and 15%, respectively. For 9 patients achieving CR after primary treatment, the median PFS was 18.0 months (range 3.0–84.0), and the 1-, 3- and 5-year PFS rates were 78, 52 and 52%, respectively. For patients with OS > 12 months and PFS > 12 months, the median ages were 21 years (range 13–35) and 17 years (range 13–35), respectively; for patients with OS < 12 months and PFS < 12 months, the median ages were 48 years (range 14–79) and 25 years (range 18–79), respectively.

Of the four patients who selected fertility-sparing surgeries, one 25-year-old woman with stage IC1 disease had a recurrence 3 months after primary treatment but achieved CR after chemotherapy. She experienced two spontaneous pregnancies and delivered twice by cesarean section [14]. Another patient, who was 16 years old with stage IC disease, received paclitaxel and carboplatin chemotherapy after primary surgery, and no tumor was detected during the subsequent 13-month follow-up period [21]. The third patient, who was 17 years old with stage IA disease, only accepted left salpingo-oophorectomy and biopsy of the right ovary, but no adjuvant therapy; she remained in CR after a follow-up of 84 months [10]. The fourth patient, who was 28 years old with unspecified-stage disease, only accepted left salpingo-oophorectomy and postoperative chemotherapy and died of recurrence 18 months after diagnosis [20].

## Discussion

PNET typically affects young people, and the most common age range is 10–19 years, followed by 20–29 years. Cases are rarely found in women over the age of 40. In our analysis, the median age of these patients was 25 years, which is far younger than the median age of epithelial ovarian tumors. Imaging studies suggest that these tumors are often solid, bulky pelvic masses, which are not specific for diagnosis. Although two of the four patients treated at PUMCH had elevated serum CA-125, there is no definitive tumor marker for PNET. Furthermore, the non-specific clinical presentations and imaging of ovarian primary PNET make early diagnosis and differential diagnosis very challenging.

IHC analysis is critical for pathological diagnosis. Sen et al. suggested that the standard diagnostic methods depend on IHC staining of markers, such as CD99 and other neural markers including NSE, synaptophysin, vimentin, S-100, NF and PZ macroglobulin [22]. Additionally, Mhawech-Fauceglia et al. suggest that PNET diagnosis shows high sensitivity and specificity when CD99 and FLI-1p are combined [23]. These authors proposed the following algorithm: (1) the diagnosis of EWS/PNET is confirmed when CD99/FLI-1p is positive in the absence of other IHC markers (except focal positivity for total cytokeratin and neuroendocrine markers) and when the fluorescence in situ hybridization (FISH) (22q12) probe is rearranged; (2) the original diagnosis of EWS/PNET should be retained when CD99/FLI-1p is positive and when (22q12) FISH presents a split signal (not rearranged); and (3) the diagnosis of EWS/PNET is excluded when CD99/FLI-1p is negative and the FISH (22q12) probe is not rearranged. No cases with a positive FISH result and CD99/FLI-1p negativity were identified.

Some authors have proposed that the diagnosis of primary ovarian PNET should be based on the presence of the EWSR1 fusion transcript and/or gene translocation detected by RT-PCR or FISH, especially when the tumor is located in an unusual site or when a diagnosis that is only based on morphology and CD99 is difficult [24]. Approximately 80–95% of ESFT patients harbored the translocation t (11;22) (q24;q12) and the consequent EWS/FLI-1 fusion gene, 5–10% carried t (21; 22) (q22; q12) and the consequent EWS/ERG gene, and less than 1% patients had t (7;22)(p22;q12), t(17;22)(q12; q12), t(2;22)(q33;q12), and inv.(22); the fused genes of EWS/ETV1, EWS/EIAF, EWS/FEV and EWS/ZSG were detected separately [25]. Therefore, detection of the EWS/FLI-1 fusion gene aids in the diagnosis of primary ovarian PNET.

No standard therapy exists for ovarian primary PNET, and surgery is still the first choice of treatment. In our analysis, two of the four patients who accepted fertility-sparing surgeries experienced relapse, whereas three patients ultimately achieved CR. One patient had two full-term deliveries. Therefore, fertility-preserving comprehensive staging surgery may be appropriate for young stage I patients, even though the risk of recurrence is high.

Due to the heterogeneity of treatment, achieving consensus regarding the regimens and courses of postoperative adjuvant chemotherapy is difficult. Initially, the chemotherapy regimen for ovarian primary PNET patients was based on the regimen for ovarian immature teratoma. Later, this tumor was regarded as a type of ovarian germ cell tumor. One 13-year-old girl diagnosed with primary ovarian PNET of at least FIGO stage IIIC received a high dose of chemotherapy and a peripheral blood progenitor cell transfusion, and there were no signs of tumor recurrence at 18 months after the completion of treatment [20]. According to the study, ESFT and VACD (vincristine, actinomycin, cyclophosphamide, doxorubicin) combined with radiotherapy can improve the 5-year tumor-free survival rate and OS rate compared with CAV (when doxorubicin reaches the lifetime dose, it should be replaced with actinomycin). Moreover, with the addition of IE (ifosfamide, etoposide) to the VACD protocol, the 5-year tumor-free survival rate and OS rate may further increased with fewer adverse effects [26].

Overall, the effectiveness of postoperative adjuvant radiotherapy is not clear. Among the 19 cases evaluated, only one patient (5.3%) received simple adjuvant radiotherapy after surgery, and three (15.8%) received a combination of chemotherapy and radiotherapy after surgery. However, these patients had poor prognoses, with survival times ranging from 2 to 17 months. ESFT, however, is to some degree sensitive to radiotherapy, and adjuvant radiotherapy has been reported to help decrease the rate of local recurrence [27]. For a tumor that cannot be removed, radiotherapy can also help reduce the tumor burden. Therefore, adjuvant radiotherapy should be administered to primary ovarian PNET patients. To avoid radiation-induced sarcoma, adoption of postoperative adjuvant radiotherapy is not recommended if radical resection of the tumor can be performed [28].

Targeted therapy of genetic mutations may provide new treatment options. A recent study has shown that EWS-FLI1 antagonists can inhibit the proliferation of ESFT cells in vitro [29]; consequently, detection of the EWS-FLI1 chimeric gene and the correct diagnosis of pPNET are of high importance. Insulin-like growth factor-1 receptor antibodies and mechanistic targets of rapamycin inhibitors have shown some clinical effect in the treatment of recurrent PNET, and these agents can also enhance patient tolerance to cancer treatment [30]. Thus, combining targeted therapy with surgery, adjuvant radiotherapy and chemotherapy may be considered for such patients. Nonetheless, there has been no valid evidence to date that targeted therapy or its combination with chemotherapy results in superior survival outcomes.

The risk factors that determine the prognoses of ovarian primary PNET patients are not clear. Due to the limited sample size, risk factors for survival could not be determined in the present study. Some authors have suggested that the prognosis is poor for those diagnosed with distant metastasis [16, 21]. Our analysis also found that even patients with stage I disease may experience rapid relapse. Regardless, the overall prognosis for patients with stage IA disease was excellent. Two patients with stage IA disease accepted fertility-sparing surgeries without any adjuvant therapy; one maintained a PFS of 84 months and the other a PFS of 36 months [10]. Dutta suggested that the prognostic factors of pPNET are tumor size, patient age and FIGO staging at diagnosis [31]. In our analysis, those with PFS times over 12 months were all young women (median age 17 years, range 13–35 years), supporting Dutta’s finding. More cases are needed to further examine high-risk factors associated with the survival of patients with ovarian primary PNET.

The most important limitation of this study was the nature of the pooled analysis, which limited the sample size, caused treatment heterogeneity, and produced a bias regarding survival data. However, the rarity of ovarian primary PNET is associated with these shortcomings. In addition, identification of EWSR1 fusion transcripts and/or gene translocation detection by FISH are/is important for the differential diagnosis of ovarian PNET [32]. Nonetheless, due to the distant storage period of pathological material, we could not perform FISH.

## Conclusion

Primary ovarian PNET is extremely rare, and the chief complaints of these patients are abdominal pain, abdominal distension and pelvic masses. The age of peak occurrence in this study was 10 to 19 years. Additionally, the gold standard diagnosis of this tumor was based on microscopic, IHC, and FISH data. No standard therapy exists to date, and most treatment options are empirical. Furthermore, the results of this study suggest that gynecological oncologists should perform comprehensive staging surgery or tumor cytoreductive surgery at the beginning, followed by postoperative adjuvant radiotherapy and combined chemotherapy (alternation between VACD and IE). Although the prognosis of patients with early-stage treatment was acceptable, the therapeutic effect for patients with metastatic disease at diagnosis remains poor. Therefore, exploration of the optimal chemotherapy regimen for ovarian primary PNET continues to be necessary.

## Data Availability

The detailed data had illustrated in the main document.

## Abbreviations

AC: Abdominal circumference
AUB: Atypical uterine bleeding
BSO: Bilateral salpingo-oophorectomy
CHEM: Chemotherapy
CRS: Cytoreductive surgery
CS: Cesarean section
CTX: Cyclophosphamide
DDP: Cisplatin
DOD: Died of disease
EP + THP: Etoposide, cisplatin, pirarubicin
EP + TPT: Etoposide, cisplatin, topotecan
EWS: Ewing’s sarcoma
IFO: Ifosfamide
KSM: Actinomycin
L: Left side
LOV: Left ovary
LPLND: Left pelvic lymph node dissection
LS: Left salpingectomy
LSO: Left salpingo-oophorectomy
M: Months
N/A: Not available
NED: No evidence of disease
OM: Omentectomy
OP: Operation
OS: Overall survival
PAC: Cisplatin, actinomycin, cyclophosphamide
PALND: Paraaortic lymph node dissection
PBPC: Peripheral Blood Progenitor Cell
PEB: Cisplatin, etoposide, bleomycin
PEI: Cisplatin, etoposide, ifosfamide
PLND: Pelvic lymph node dissection
PUMCH: Peking Union Medical College Hospital
PVB: Cisplatin, vincristine, bleomycin
R: Right side
RAD: Radiotherapy
RCRS: Recytoreductive surgery
ROV: Right ovary
RSO: Right salpingo-oophorectomy
TAH: Total abdominal hysterectomy
TC: Paclitaxel, carboplatin
VAC: Vincristine, actinomycin, cyclophosphamide
VACA: Vincristine, actinomycin, cyclophosphamide, doxorubicin
VCD: Vincristine, cyclophosphamide, doxorubicin
VCR: Vincristine
VIA: Vincristine, ifosfamide and actinomycin
VID: Vincristine, ifosfamide and doxorubicin
VIE: Vincristine, ifosfamide, etoposide
VIP: Vincristine, ifosfamide, cisplatin
VP16: Etoposide
Y: Years
